# Intestinal obstruction due to small bowel herniation through foramen of Winslow: a case report 

**DOI:** 10.1186/s13256-020-02629-w

**Published:** 2021-01-15

**Authors:** P. K. B. S. C. Bandara, A. M. Viraj Rohana, Aloka Pathirana

**Affiliations:** 1 Colombo South Teaching Hospital, Kalubowila, Sri Lanka; 2 Faculty of Medical Sciences, University of Sri Jayawardenapura, Nugegoda, Sri Lanka

**Keywords:** Case report, Internal hernia, Foramen of Winslow, Intestinal obstruction, Small bowel herniation

## Abstract

**Background:**

Intestinal obstruction due to internal herniation of the bowel is a rare clinical entity which is often overlooked in the differential diagnosis of patients with abdominal pain who have no previous history of abdominal surgery. Several sites of bowel internal herniation have been described, amongst which internal herniation through the foramen of Winslow accounts for about 8% of cases. These patients present with nonspecific abdominal pain associated with symptoms of gastroesophageal reflux disease, and hence the diagnosis is often overlooked. The usual symptoms of intestinal obstruction can be delayed, which results in a delay in diagnosis and gangrene of the herniated bowel segment. Abdominal radiographs and computed tomography are helpful in the diagnosis. Open reduction is the management of choice; however, laparoscopic reduction has also been attempted, with good results.

**Case presentation:**

We report a case of a middle-aged Sri Lankan man who presented with features of gastroesophageal reflux disease, developed features of intestinal obstruction and was found to have a gangrenous small bowel loop which had herniated through the foramen of Winslow. Following needle aspiration and reduction of the herniated small bowel loop, the gangrenous part of the small bowel was resected and an ileoileal anastomosis performed. The large foramen of Winslow was partially closed with interrupted stitches. The patient made an uneventful recovery.

**Conclusion:**

Since delayed diagnosis of bowel obstruction is detrimental, it is of utmost importance to diagnose it early. Because internal herniation of the small bowel through the foramen of Winslow presents with nonspecific symptoms including features of gastroesophageal reflux disease, as documented in several cases worldwide and also presented by our patient, there should be a high degree of suspicion of internal herniation of the bowel causing bowel obstruction and low threshold for extensive investigation of patients presenting with symptoms of gastroesophageal reflux disease which does not resolve with usual medication.

## Introduction

Internal hernias are a rare clinical entity, accounting for 0.2–0.9% of all hernias. The incidence of intestinal obstruction due to internal herniation of the bowel is 0.6–5.8% [1]. Internal herniation of the bowel can be paraduodenal, transmesenteric, pericecal, through the foramen of Winslow or omental. Of these, herniation through the foramen of Winslow accounts for about 8% of all internal hernias [2]. We report a patient presenting with nonspecific abdominal pain and symptoms of gastroesophageal reflux disease who was later found to have a necrotic segment of the small bowel which had herniated through the foramen of Winslow.

## Case presentation

**Fig. 1:**
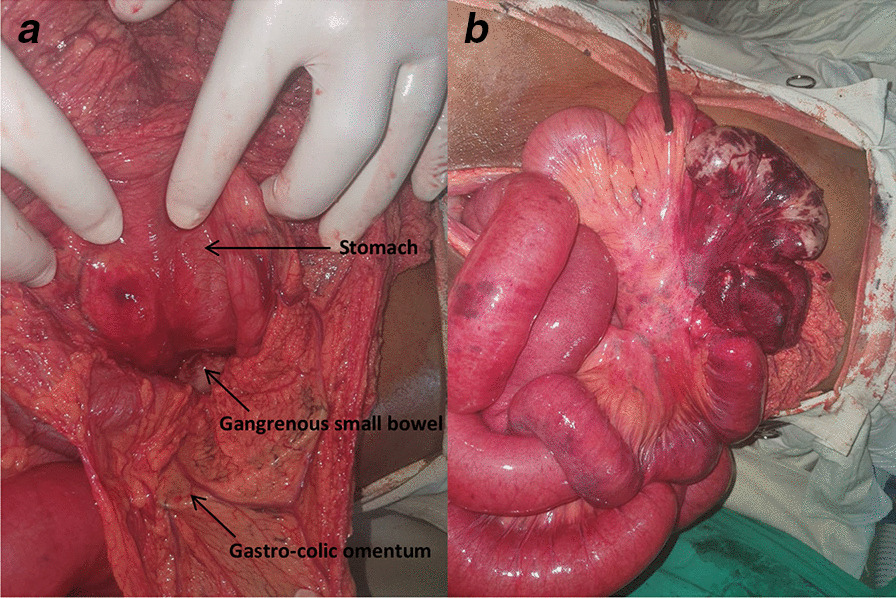
**a** Lesser sac opened through the gastrocolic omentum. **b** Gangrenous part of the bowel

**Fig. 2:**
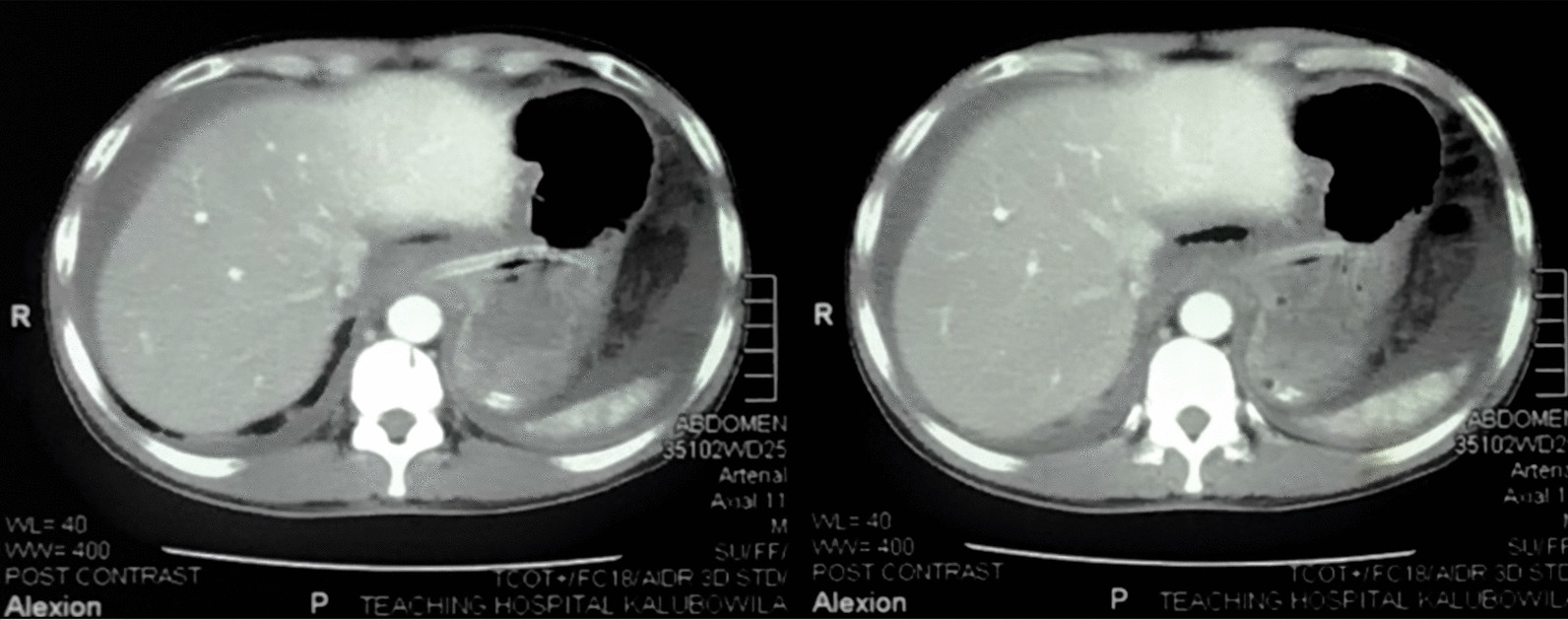
Contrast-enhanced computed tomography of the abdomen showing dilated small bowel loops

A healthy 52-year-old Sri Lankan man with no previous medical or surgical history presented with epigastric pain associated with several episodes of bilious vomiting for 1 day. Bowel functions were normal. He was afebrile, there was mild epigastric tenderness, and the digital rectal examination was normal. He was initially treated with analgesics, prokinetics and proton pump inhibitors. His symptoms worsened, and on the third day he complained of abdominal distention and persistent vomiting. Increasing epigastric tenderness with gaseous abdominal distention was noted, and he had not had bowel movement since admission. He was hemodynamically stable but had elevated inflammatory markers (white blood cell count 17 × 10^3^/l and C-reactive protein 139 mg/l), and abdominal X-ray revealed dilated small bowel loops. Ultrasound scan of the abdomen was normal. Urgent contrast-enhanced computed tomography revealed dilated small bowel loops without an obvious cause of obstruction. The patient underwent emergency exploratory laparotomy and was found to have a gangrenous mid-ileal bowel loop herniating through a tight foramen of Winslow (Fig. 1). Since it was difficult to reduce the gangrenous segment through the foramen of Winslow, the lesser sac was opened through the gastrocolic omentum and needle decompression of the dilated gangrenous bowel loop was performed before reduction. Segmental resection (of the gangrenous part) and a side-to-side ileoileal anastomosis was performed. The enlarged defect of the foramen of Winslow was closed with interrupted stitches. The patient made an uneventful recovery and was discharged on postoperative day 6. Follow-up 1 month after surgery was uneventful (Fig. 2).

## Discussion

Internal hernias are a rare clinical entity, accounting for 0.2–0.9% of all cases of intestinal obstruction, amongst which herniation through the foramen of Winslow is very rare, constituting only 8% of internal hernias [3]. The small bowel is most often involved, followed by the cecum and right colon and then the transverse colon [2]. Peculiar cases of herniated Meckel’s diverticulum and gallbladder have also been reported [4, 5]. Postulated predisposing factors for this condition include atrophic greater omentum, short transverse mesocolon, long mesentery of the small intestine, and abnormalities of intestinal rotation and a large foramen of Winslow [6]. This patient was found to have a large foramen of Winslow, which we partially closed.

As internal herniation through the foramen of Winslow often presents with nonspecific symptoms such as epigastric pain, regurgitation, nausea and vomiting, differential diagnoses range from gastrointestinal to pancreatic or gallbladder pathology [7]. The reason for these nonspecific symptoms could be the compression of the stomach by the herniated bowel loop through the foramen of Winslow.

The diagnosis is made either preoperatively or intraoperatively. The presence of a circumscribed collection of bowel gas with air fluid levels high up in the left upper abdomen and displacement of the gastric air bubble to the left in plain abdominal radiographs is suggestive of herniation of the small bowel through the foramen of Winslow [8, 9]. Computed tomographic features include an air fluid collection in the lesser sac, with a beak directed towards the foramen of Winslow between the pancreas and the stomach anterior to the inferior vena cava and posterior to the liver hilum. Evidence of intestinal obstruction associated with the presence of mesenteric vessels stretching anterior to the vena cava and posterior to the portal vein is another feature noted in the CT [9].

Management involves reduction of the hernia either laparoscopically or by a laparotomy before the bowel becomes gangrenous [3]. Gentle traction is sufficient to reduce the hernia most of the time. There have been instances where opening of the gastrocolic or gastrohepatic ligaments or even a Kocher maneuver was necessary to reduce the hernia [3]. When reduction is difficult, needle or catheter decompression of the incarcerated intestine has been tried in situ within the lesser sac [6]. Once reduced, the gangrenous segment of the bowel (if present) requires resection and anastomosis [3]. Closure of the foramen of Winslow in order to prevent recurrence is doubtful, as no recurrence in cases without closure of the defect has been reported to date, and an attempt to close the defect also risks damaging the vital structures around the area [3, 6].

## Conclusion

Delayed diagnosis of small bowel obstruction results in serious complications including perforation, gross peritoneal contamination, sepsis and death. As our patient was diagnosed on the third day following hospital admission, he had a gangrenous bowel segment which had to be resected. The primary reason for the delay in diagnosis was that the patient complained of an epigastric pain with regurgitation which we attributed to gastroesophageal reflux disease. In retrospect, the symptoms were likely brought about by compression of the stomach by the congested, oedematous herniated small bowel loop. Hence suspicion of a sinister pathology is important in a patient who presents with unresolving epigastric pain suggestive of gastroesophageal reflux disease even in the absence of other symptoms, and highlights the importance of early computed tomography followed by early surgical intervention to prevent further complications.

### Patient perspective

At the 1-month review of the patient, he is thankful for the treatments he received from the admission until the discharge and not worried about the delayed diagnosis given the complexity of his presentation. However, being a manual worker is he worried about not being able to carry out his occupation to the fullest of ability yet, and he attributes it to the mild pain he experiences at the surgical site from time to time.

## Data Availability

Not applicable.
